# 4,4′-Bipyridine–*trans*,*trans*-hexa-2,4-dienedioic acid (1/1)

**DOI:** 10.1107/S1600536812012391

**Published:** 2012-03-28

**Authors:** Suk-Hee Moon, Ki-Min Park

**Affiliations:** a Department of Food and Nutrition, Kyungnam College of Information and Technology, Busan 617-701, Republic of Korea; b Department of Chemistry and Research Institute of Natural Sciences, Gyeongsang, National University, Jinju 660-701, Republic of Korea

## Abstract

The title cocrystal, C_10_H_8_N_2_·C_6_H_6_O_4_, crystallizes with half-mol­ecules of 4,4′-bipyridine and *trans*,*trans*-hexa-2,4-dienedioic acid in the asymmetric unit, as each is located about a crystallographic inversion center. The bipyridine molecule is planar from symmetry. In the dicarboxylic acid molecule, the O—C—C—C torsion angle is −13.0 (2)°. In the crystal, O—H⋯N and C—H⋯O hydrogen bonds generate a three-dimensional network.

## Related literature

For cocrystals of carb­oxy­lic acid and pyridine, see: Bhogala & Nangia (2003 ▶); Hou *et al.* (2008 ▶); Jiang & Hou (2012 ▶). For background to the applications of cocrystals, see: Bhogala & Nangia (2003 ▶); Gao *et al.* (2004 ▶); Hori *et al.* (2009 ▶); Weyna *et al.* (2009 ▶).
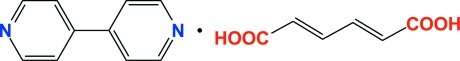


## Experimental

### 

#### Crystal data

C_10_H_8_N_2_·C_6_H_6_O_4_
*M* *_r_* = 298.29
Triclinic, 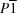

*a* = 5.8481 (5) Å
*b* = 7.6348 (6) Å
*c* = 8.4677 (7) Å
α = 91.837 (5)°
β = 92.584 (5)°
γ = 111.907 (4)°
*V* = 349.93 (5) Å^3^
*Z* = 1
Mo *K*α radiation
μ = 0.10 mm^−1^
*T* = 173 K
0.40 × 0.26 × 0.24 mm

#### Data collection

Bruker APEXII CCD diffractometer
Absorption correction: multi-scan (*SADABS*; Sheldrick, 1996 ▶) *T*_min_ = 0.960, *T*_max_ = 0.976
5973 measured reflections
1517 independent reflections
1336 reflections with *I* > 2σ(*I*)
*R*_int_ = 0.031

#### Refinement

*R*[*F* ^2^ > 2σ(*F* ^2^)] = 0.040
*wR*(*F* ^2^) = 0.116
*S* = 1.04
1517 reflections
104 parameters
H atoms treated by a mixture of independent and constrained refinement
Δρ_max_ = 0.24 e Å^−3^
Δρ_min_ = −0.25 e Å^−3^

### 

Data collection: *APEX2* (Bruker, 2006 ▶); cell refinement: *SAINT* (Bruker, 2006 ▶); data reduction: *SAINT*; program(s) used to solve structure: *SHELXTL* (Sheldrick, 2008 ▶); program(s) used to refine structure: *SHELXTL*; molecular graphics: *SHELXTL* and *DIAMOND* (Brandenburg, 1998 ▶); software used to prepare material for publication: *SHELXTL*.

## Supplementary Material

Crystal structure: contains datablock(s) I, global. DOI: 10.1107/S1600536812012391/sj5219sup1.cif

Structure factors: contains datablock(s) I. DOI: 10.1107/S1600536812012391/sj5219Isup2.hkl

Supplementary material file. DOI: 10.1107/S1600536812012391/sj5219Isup3.cml

Additional supplementary materials:  crystallographic information; 3D view; checkCIF report

## Figures and Tables

**Table 1:** Hydrogen-bond geometry (Å, °)

*D*—H⋯*A*	*D*—H	H⋯*A*	*D*⋯*A*	*D*—H⋯*A*
O1—H1⋯N1	1.06 (2)	1.58 (2)	2.6148 (14)	164 (2)
C2—H2⋯O2^i^	0.95	2.65	3.5130 (15)	151
C3—H3⋯O2^ii^	0.95	2.58	3.4457 (16)	152
C4—H4⋯O1^iii^	0.95	2.58	3.4848 (17)	160
C8—H8⋯O2^iv^	0.95	2.56	3.3555 (17)	141

Symmetry codes: (i) 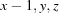; (ii) 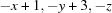; (iii) 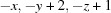; (iv) 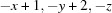.

**Fig. 1.**
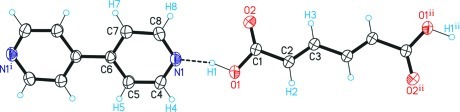
The molecular structure of the title compound, showing displacement ellipsoids drawn at the 50% probability level. Hydrogen bonds are shown as dashed lines. (Symmetry codes: i) -x + 1, -y + 1, -z + 1; ii) -x, -y + 3, -z.)

**Fig. 2.**
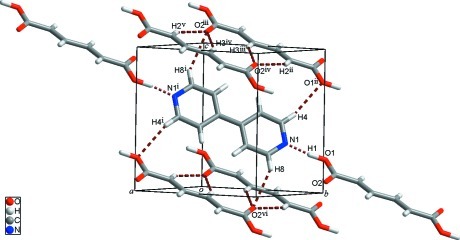
Crystal packing of the title compound with intermolecular O—H···N and C—H···O hydrogen bonds shown as dashed lines. (Symmetry codes: i) -x + 1, -y + 1, -z + 1; ii) -x, -y + 2, -z + 1; iii) x, y - 1, z + 1; iv) -x + 1, -y + 2, -z + 1; v) x + 1, y + 1, z + 1; vi) -x + 1, -y + 2, -z.)

## supplementary crystallographic information

### Comment

Considerable effort has been devoted to form co-crystals made up of two or more components because of their potential applications in pharmaceutical chemisty (Weyna *et al.*, 2009), supramolecular chemistry (Bhogala & Nangia, 2003; Gao *et al.*, 2004) and materials chemistry (Hori *et al.*, 2009). In particular, numerous studies have focused on hydrogen bonding between carboxylic acid and pyridine molecules (Bhogala & Nangia, 2003; Hou *et al.*, 2008; Jiang & Hou, 2012). We report here the structure of a co-crystal of *trans*, *trans*-hexa-2,4-dienedioic acid with 4,4'-bipyridine in the solid state.

The title compound is shown in Fig. 1. The asymmetric unit contains half-molecules of 4,4'-bipyridine and *trans*,*trans*-1,3 -butadiene-1,4-dicarboxylic acid each located on crystallographic inversion centers. Both components are planar by symmetry and tilted by 32.02 (7)° with respect to each other.

In the crystal, the dicarboxylic acid molecules are arranged side by side by intermolecular C—H···O hydrogen bonds between the dicarboxylic acid molecules, leading to the formation of a one dimensional chain. Moreover, intermolecular O—H···N and C—H···O hydrogen bonds between dicarboxylic acid and 4,4'-bipyridine molecules generate a three-dimensional network (Fig. 2, Table 1).

### Experimental

A mixture of stoichiometric amounts of *trans*, *trans*-hexa-2,4-dienedioic acid and 4,4'-bipyridine in DMF (in a 1:1 volume ratio) was heated until the two components dissolved and was then kept at room temperature. Upon slow evaporation of the solvent, *X*–ray quality single crystals were obtained.

### Refinement

The carboxyl-H atom was located in a difference Fourier map and refined isotropically. C*sp*^2^ H atoms were positioned geometrically and refined using a riding model with d(C—H) = 0.95 Å and *U*_iso_(H) = 1.2*U*_eq_(C).

### Crystal data

**Table:**

C_10_H_8_N_2_·C_6_H_6_O_4_	*Z* = 1
*M**_r_* = 298.29	*F*(000) = 156
Triclinic, *P*1	*D*_x_ = 1.415 Mg m^−^^3^
Hall symbol: -P 1	Mo *K*α radiation, λ = 0.71073 Å
*a* = 5.8481 (5) Å	Cell parameters from 4151 reflections
*b* = 7.6348 (6) Å	θ = 2.4–28.4°
*c* = 8.4677 (7) Å	µ = 0.10 mm^−^^1^
α = 91.837 (5)°	*T* = 173 K
β = 92.584 (5)°	Block, colourless
γ = 111.907 (4)°	0.40 × 0.26 × 0.24 mm
*V* = 349.93 (5) Å^3^	

### Data collection

**Table:**

Bruker APEXII CCD diffractometer	1517 independent reflections
Radiation source: fine-focus sealed tube	1336 reflections with *I* > 2σ(*I*)
Graphite monochromator	*R*_int_ = 0.031
φ and ω scans	θ_max_ = 27.0°, θ_min_ = 2.4°
Absorption correction: multi-scan (*SADABS*; Sheldrick, 1996)	*h* = −7→7
*T*_min_ = 0.960, *T*_max_ = 0.976	*k* = −9→9
5973 measured reflections	*l* = −10→10

### Refinement

**Table:**

Refinement on *F*^2^	Primary atom site location: structure-invariant direct methods
Least-squares matrix: full	Secondary atom site location: difference Fourier map
*R*[*F*^2^ > 2σ(*F*^2^)] = 0.040	Hydrogen site location: inferred from neighbouring sites
*wR*(*F*^2^) = 0.116	H atoms treated by a mixture of independent and constrained refinement
*S* = 1.04	*w* = 1/[σ^2^(*F*_o_^2^) + (0.0688*P*)^2^ + 0.0753*P*] where *P* = (*F*_o_^2^ + 2*F*_c_^2^)/3
1517 reflections	(Δ/σ)_max_ < 0.001
104 parameters	Δρ_max_ = 0.24 e Å^−^^3^
0 restraints	Δρ_min_ = −0.25 e Å^−^^3^

### Special details

**Table:**

Geometry. All e.s.d.'s (except the e.s.d. in the dihedral angle between two l.s. planes) are estimated using the full covariance matrix. The cell e.s.d.'s are taken into account individually in the estimation of e.s.d.'s in distances, angles and torsion angles; correlations between e.s.d.'s in cell parameters are only used when they are defined by crystal symmetry. An approximate (isotropic) treatment of cell e.s.d.'s is used for estimating e.s.d.'s involving l.s. planes.
Refinement. Refinement of *F*^2^ against ALL reflections. The weighted *R*-factor *wR* and goodness of fit *S* are based on *F*^2^, conventional *R*-factors *R* are based on *F*, with *F* set to zero for negative *F*^2^. The threshold expression of *F*^2^ > σ(*F*^2^) is used only for calculating *R*-factors(gt) *etc*. and is not relevant to the choice of reflections for refinement. *R*-factors based on *F*^2^ are statistically about twice as large as those based on *F*, and *R*- factors based on ALL data will be even larger.

### Fractional atomic coordinates and isotropic or equivalent isotropic displacement parameters (Å^2^)

**Table:**

	*x*	*y*	*z*	*U*_iso_*/*U*_eq_	
O1	0.05949 (18)	1.05053 (14)	0.23387 (13)	0.0414 (3)	
H1	0.184 (4)	0.987 (3)	0.271 (3)	0.084 (7)*	
O2	0.37461 (17)	1.23144 (14)	0.09959 (12)	0.0394 (3)	
C1	0.1653 (2)	1.18769 (18)	0.13952 (14)	0.0285 (3)	
C2	−0.0033 (2)	1.28402 (18)	0.09016 (15)	0.0299 (3)	
H2	−0.1740	1.2262	0.1077	0.036*	
C3	0.0786 (2)	1.44912 (17)	0.02208 (14)	0.0288 (3)	
H3	0.2485	1.5031	0.0013	0.035*	
N1	0.3031 (2)	0.85144 (15)	0.34658 (13)	0.0331 (3)	
C4	0.2266 (3)	0.7745 (2)	0.48325 (17)	0.0374 (3)	
H4	0.1161	0.8150	0.5389	0.045*	
C5	0.3007 (3)	0.6385 (2)	0.54775 (16)	0.0350 (3)	
H5	0.2419	0.5882	0.6458	0.042*	
C6	0.4615 (2)	0.57572 (16)	0.46878 (14)	0.0262 (3)	
C7	0.5464 (3)	0.6613 (2)	0.32875 (16)	0.0351 (3)	
H7	0.6611	0.6270	0.2719	0.042*	
C8	0.4627 (3)	0.79683 (19)	0.27252 (16)	0.0362 (3)	
H8	0.5224	0.8532	0.1766	0.043*	

### Atomic displacement parameters (Å^2^)

**Table:**

	*U*^11^	*U*^22^	*U*^33^	*U*^12^	*U*^13^	*U*^23^
O1	0.0346 (5)	0.0401 (6)	0.0593 (7)	0.0222 (4)	0.0127 (5)	0.0259 (5)
O2	0.0314 (5)	0.0469 (6)	0.0488 (6)	0.0229 (4)	0.0109 (4)	0.0174 (5)
C1	0.0283 (6)	0.0293 (6)	0.0319 (6)	0.0148 (5)	0.0032 (5)	0.0056 (5)
C2	0.0260 (6)	0.0327 (7)	0.0355 (6)	0.0156 (5)	0.0037 (5)	0.0077 (5)
C3	0.0270 (6)	0.0324 (6)	0.0316 (6)	0.0156 (5)	0.0042 (5)	0.0067 (5)
N1	0.0322 (6)	0.0287 (6)	0.0407 (6)	0.0141 (5)	−0.0015 (5)	0.0074 (4)
C4	0.0397 (7)	0.0387 (7)	0.0434 (7)	0.0248 (6)	0.0073 (6)	0.0078 (6)
C5	0.0406 (7)	0.0390 (7)	0.0339 (7)	0.0234 (6)	0.0080 (5)	0.0106 (5)
C6	0.0253 (6)	0.0255 (6)	0.0291 (6)	0.0110 (5)	−0.0015 (5)	0.0030 (5)
C7	0.0372 (7)	0.0377 (7)	0.0373 (7)	0.0205 (6)	0.0084 (5)	0.0114 (6)
C8	0.0395 (7)	0.0351 (7)	0.0382 (7)	0.0176 (6)	0.0055 (6)	0.0135 (6)

### Geometric parameters (Å, º)

**Table:**

O1—C1	1.3162 (15)	C4—C5	1.3839 (18)
O1—H1	1.06 (2)	C4—H4	0.9500
O2—C1	1.2096 (16)	C5—C6	1.3907 (17)
C1—C2	1.4873 (16)	C5—H5	0.9500
C2—C3	1.3310 (18)	C6—C7	1.3929 (18)
C2—H2	0.9500	C6—C6^ii^	1.492 (2)
C3—C3^i^	1.452 (2)	C7—C8	1.3872 (17)
C3—H3	0.9500	C7—H7	0.9500
N1—C8	1.3286 (17)	C8—H8	0.9500
N1—C4	1.3337 (18)		
			
C1—O1—H1	110.3 (13)	C5—C4—H4	118.5
O2—C1—O1	124.23 (11)	C4—C5—C6	119.86 (12)
O2—C1—C2	124.24 (11)	C4—C5—H5	120.1
O1—C1—C2	111.52 (10)	C6—C5—H5	120.1
C3—C2—C1	121.76 (12)	C5—C6—C7	116.57 (11)
C3—C2—H2	119.1	C5—C6—C6^ii^	121.53 (14)
C1—C2—H2	119.1	C7—C6—C6^ii^	121.90 (14)
C2—C3—C3^i^	123.31 (15)	C8—C7—C6	119.78 (12)
C2—C3—H3	118.3	C8—C7—H7	120.1
C3^i^—C3—H3	118.3	C6—C7—H7	120.1
C8—N1—C4	117.62 (11)	N1—C8—C7	123.05 (12)
N1—C4—C5	123.05 (12)	N1—C8—H8	118.5
N1—C4—H4	118.5	C7—C8—H8	118.5
			
O2—C1—C2—C3	−13.0 (2)	C4—C5—C6—C6^ii^	−177.69 (14)
O1—C1—C2—C3	166.14 (12)	C5—C6—C7—C8	−2.2 (2)
C1—C2—C3—C3^i^	−177.53 (14)	C6^ii^—C6—C7—C8	177.72 (14)
C8—N1—C4—C5	−1.8 (2)	C4—N1—C8—C7	1.8 (2)
N1—C4—C5—C6	−0.3 (2)	C6—C7—C8—N1	0.2 (2)
C4—C5—C6—C7	2.3 (2)		

Symmetry codes: (i) −*x*, −*y*+3, −*z*; (ii) −*x*+1, −*y*+1, −*z*+1.

### Hydrogen-bond geometry (Å, º)

**Table:**

*D*—H···*A*	*D*—H	H···*A*	*D*···*A*	*D*—H···*A*
O1—H1···N1	1.06 (2)	1.58 (2)	2.6148 (14)	164 (2)
C2—H2···O2^iii^	0.95	2.65	3.5130 (15)	151
C3—H3···O2^iv^	0.95	2.58	3.4457 (16)	152
C4—H4···O1^v^	0.95	2.58	3.4848 (17)	160
C8—H8···O2^vi^	0.95	2.56	3.3555 (17)	141

Symmetry codes: (iii) *x*−1, *y*, *z*; (iv) −*x*+1, −*y*+3, −*z*; (v) −*x*, −*y*+2, −*z*+1; (vi) −*x*+1, −*y*+2, −*z*.
